# Possible Mechanism of SARS-CoV-2 Nsp1-Mediated Control of Viral Gene Expression

**DOI:** 10.3389/fcimb.2022.881749

**Published:** 2022-04-22

**Authors:** Tie Zhao, Yi Ren, Jianguo Wu, Qiwei Zhang

**Affiliations:** ^1^ Guangdong Provincial Key Laboratory of Virology, Institute of Medical Microbiology, Jinan University, Guangzhou, China; ^2^ Institute of Pathogenic Biology and Key Laboratory of Special Pathogen Prevention and Control of Hunan Province, Hengyang Medical College, University of South China, Hengyang, China; ^3^ Foshan Institute of Medical Microbiology, Foshan, China; ^4^ BSL-3 Laboratory (Guangdong), Guangdong Provincial Key Laboratory of Tropical Disease Research, School of Public Health, Southern Medical University, Guangzhou, China

**Keywords:** SARS-CoV-2, mechanism, Nsp1, translational suppression, mRNA

## Highlights

- The relation between the structures of Nsp1 proteins and their function is further revealed.
- The molecular mechanism of viral mRNA escape from Nsp1 suppression is developed and interpreted.
- The Nsp1 has no effect on viral proteins, which is helpful to gain insights into SARS-CoV-2 infection and to understand the impacts of protein structure in the context of replication.

Recent studies have confirmed the key role of non-structural protein 1 (Nsp1) of severe acute respiratory syndrome coronavirus 2 (SARS-CoV-2) in the pathogenesis of COVID-19. This protein suppresses host gene expression and especially blocks the interferon expression, while it permits the translation of viral mRNA (Banerjee et al., 2020; Schubert et al., 2020; Thoms et al., 2020; Finkel et al., 2021; Kumar et al., 2021).

The genera of CoVs have high similarity in the core structure (Nakagawa and Makino, 2021). Based on RNA sequencing, ribosome profiling, and the mark technique, it was found that SARS-CoV-2 controlled host protein production by a multipronged strategy (Finkel et al., 2021). Nsp1 can stall mRNA translation and lead to accelerated degradation of cellular mRNA (Kamitani et al., 2009). Interestingly and surprisingly, however, the Nsp1-mediated block of mRNA translation has no effect on viral proteins, and no one knew why.

One explanation may be that Nsp1 can reduce global cellular translation, while viral mRNAs are still translated by the remaining ribosomes (Schubert et al., 2020). However, this interpretation is incomplete and non-specific. Nsp1 can lead to the inactivation of the translation functions of 40S ribosomes (Narayanan et al., 2008). The explanation stands only if the abundance of 40S ribosomal subunits exceeds the Nsp1 protein.

The other evidence suggests that viral mRNAs with the 5′ leader are critical for viral translation (Kim et al., 2020; Finkel et al., 2021; Miao et al., 2021), or to be more precise, the key element is an RNA hairpin SL1 in the 5′ UTR of the viral genome (Tidu et al., 2020). The sequence or conformation of the secondary structure may affect the mRNA translation or not.

The SL1 hairpin as a *cis*-acting element induces structural rearrangement of Nsp1 and whether to free the access to the mRNA channel and regulate the translation of viruses or cells (Tidu et al., 2020). Other research groups also presented a similar model to suppress host defenses (Banerjee et al., 2020). The above modeling has suggested that the apical part of SL1 is a critical position for Nsp1 inhibition. Nsp1-mediated inhibition is abrogated, while the apical part of SL1 is opened by the introduction of four mutations (U, C, C, A to A, G, G, U) (Tidu et al., 2020). The accuracy of the apical part of the SL1 sequence is really important.

Cryoelectronic microscopy found that the C-terminal of Nsp1 and mRNA entry tunnel of the ribosome combined so strongly that protein synthesis was forbidden (Schubert et al., 2020; Thoms et al., 2020). There is a dynamic competitive relationship between Nsp1 and mRNA during binding of the ribosome. The Nsp1 of SARS-CoV-2 combined with the host 40S ribosomal subunit with high affinity, and the Nsp1–40S complexes disrupt cap-dependent translation (Thoms et al., 2020).

**Figure 1:**
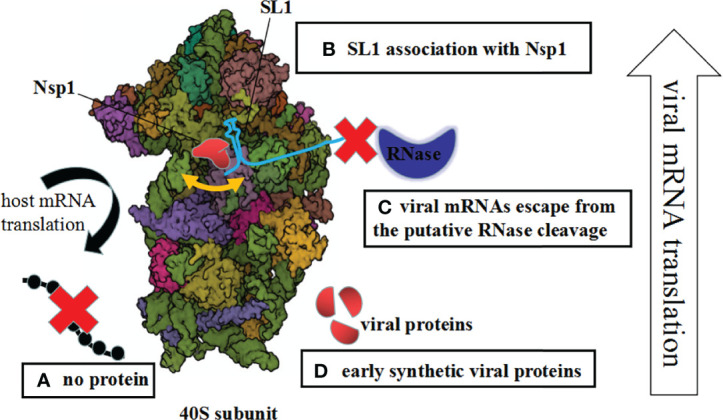
Mechanisms of Nsp1-mediated control of the host and viral gene expression. **(A)** The host mRNA translation is inhibited due to blocking of the access to mRNAs. **(B)** Upon interaction between Nsp1 and 5′ UTR of the SARS-CoV-2 genome, the mRNA channel is opened. **(C)** The unusual Nsp1–40S complex cannot interact with viral mRNAs, so the viral mRNAs escape from the putative RNase cleavage. **(D)** Early synthetic viral proteins are enough to drive the virus replication.

Actually, the Nsp1–40S complexes include idle Nsp1–40S complexes and pre-40S-like complexes with unusual 43S preinitiation complexes (Thoms et al., 2020). The available data make it reasonable to assume that Nsp1 avoids inhibiting viral proteins (**Figure 1**).

First, upon the interaction between the Nsp1 and the 5′ UTR of the SARS-CoV-2 genome, or the change of the Nsp1, ribosomal association with Nsp1 was inhibited. This is an either–or situation (Yuan et al., 2020; Lapointe et al., 2021). Previous research proved that the NSP1 and the 5′ UTR are linked functionally and interacted cooperatively with highly conserved elements of the ribosomal machinery (Sosnowski et al., 2022). Additionally, this process is complicated because Nsp1 regulates the viral-to-host translation ratio rather than simply promoting or inhibiting translation (Vora et al., 2022). Second, the complex of Nsp1 and 40S ribosomes or virus infection may generate structural alterations of the 40S ribosome. Subsequently, the unusual Nsp1–40S complex cannot interact with viral mRNAs. Therefore, the viral mRNAs escape from the putative RNase cleavage by Nsp1 suppression. What is really interesting is that nucleases during the Nsp1 interactions were identified (Bujanic et al., 2022). Last but not least, Nsp1 restrains partly virus replication; even so, early synthetic viral proteins are enough to drive the virus replication *via* the time phase of protein expression (**Figure 1**).

The above inference interprets the possible molecular mechanism of viral mRNAs escaping from Nsp1 suppression (**Figure 1**). However, the effect of Nsp1 on the translation of cellular or viral mRNA needs to be further studied, as this is a complicated dynamic process.

## Author Contributions

TZ and YR wrote the manuscript. JW and QZ performed data analysis and revised the manuscript. All authors contributed to the article and approved the submitted version.

## Funding

This work was supported by grants from the National Key Research and Development Program of China (2018YFE0204503), National Natural Science Foundation of China (32170139 and 81730061), Natural Science Foundation of Guangdong Province (2018B030312010, 2021A1515010788 and 2022A1515011190) and the Fundamental Research Funds for the Central Universities (21622101).

## Conflict of Interest

The authors declare that the research was conducted in the absence of any commercial or financial relationships that could be construed as a potential conflict of interest.

## Publisher’s Note

All claims expressed in this article are solely those of the authors and do not necessarily represent those of their affiliated organizations, or those of the publisher, the editors and the reviewers. Any product that may be evaluated in this article, or claim that may be made by its manufacturer, is not guaranteed or endorsed by the publisher.
